# Supplementary material for: Proteome profiles of esophageal squamous cell carcinoma tie mitochondrial complex I to immunotherapy
Source: EMBO Mol Med. 2026 Apr 11;18(5):1884–932. doi: 10.1038/s44321-026-00413-9 (PMC13179373; doi:10.1038/s44321-026-00413-9)
Supplement: Supplementary file 16 — Expanded View Figures [file 44321_2026_413_MOESM16_ESM.pdf]

Expanded View Figures

Figure EV1. The protein identification and quality control using the mass spectrometry platform.

(**A**) The representative images of immunohistochemistry (IHC) staining of PD-L1 expression in sensitive (S) and non-sensitive (NS) patients of immunotherapy. (**B**, **C**) The association of PD-L1 (CD274) mRNA expression with cancer immunotherapy response in the GSE78220 melanoma cohort (**B**) and IMvigor210 metastatic urothelial carcinoma cohort (**C**). Boxplots showing the PD-L1 expression difference between S and NS groups. Boxplots show median (central line), upper and lower quartiles (box limits), and minimum or maximum (whiskers) (left). The Kaplan–Meier curves of the expression of PD-L1 with overall survival (OS) (right). *P* value from two-sided log-rank test. GSE78220 melanoma cohort: $n = 15$ (S) and 13 (NS). IMvigor210 metastatic urothelial carcinoma cohort: $n = 68$ (S) and 230 (NS). (**D**) The Kaplan–Meier plots of S and NS patients of ESCC in OS and PFS in the MSK-IMPACT cohort (two-sided log-rank test). (**E**) The association of TMB level with OS and PFS in the MSK-IMPACT cohort (two-sided log-rank test). (**F**) The association of TMB level with OS in the TCGA-ESCC cohort (two-sided log-rank test). (**G**) Longitudinal quality control of MS using tryptic digests of HEK293T cells (left) and ESCC samples pool (right). The bottom-left half of the panel represents the pairwise Pearson's correlation coefficients of the samples (two-sided Pearson's correlation test), and the top-right half of the panel depicts the pairwise scatter plots from the same comparison. (**H**) The proteome and phosphoproteome identification between the S and NS groups. Venn diagrams show the overlap of proteins, phosphoproteins, and phosphosites in S and NS groups (upper). Barplots display the identified number of proteins, phosphoproteins, and phosphosites (bottom). *P* value from two-sided Student's *t* test. $n = 24$ (S) and 29 (NS). Data are presented as mean with standard error of the mean. (**I**) Boxplot for $\log_{10}$(FOT) of proteins (upper) and phosphoproteins (bottom) in 53 ESCC patients. Boxplots show median (central line), upper and lower quartiles (box limits), and 1.5 × interquartile range (whiskers). (**J**) Principal components analysis (PCA) of proteins (left) and phosphoproteins (right) levels in 53 ESCC patients. (**K**) The batch effects evaluation of the proteomic (left) and phosphoproteomic (right) data during MS detection. The *x* axis showed the principal components. The right *y* axis (for the scatter plot) indicates the explained ratio of principal components. The left *y* axis (for the barplot) was the Spearman correlation of batch effects variable and each principal component.

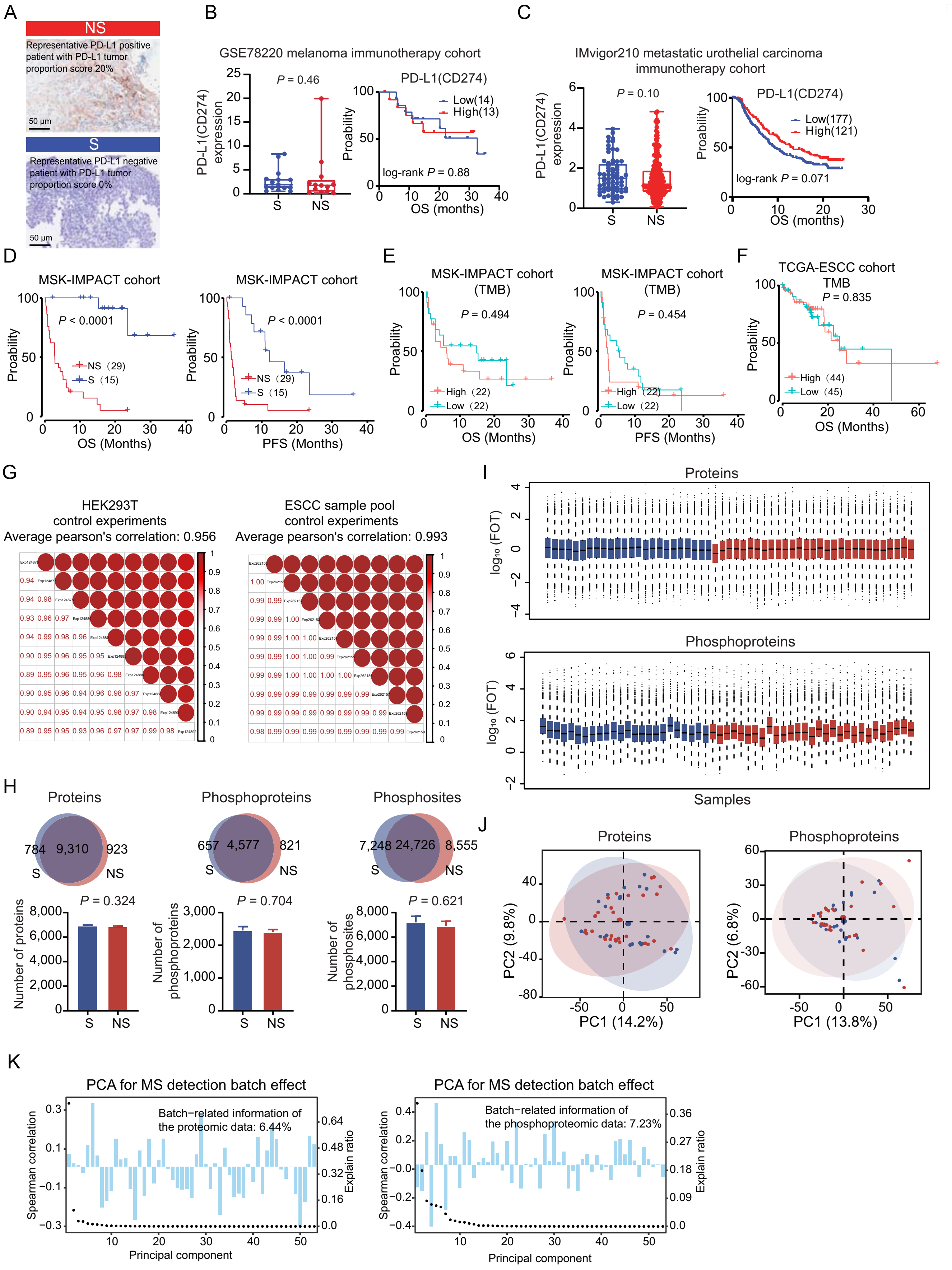
A
NS
Representative PD-L1 positive patient with PD-L1 tumor proportion score 20%
50 μm
S
Representative PD-L1 negative patient with PD-L1 tumor proportion score 0%
50 μm
B
GSE78220 melanoma immunotherapy cohort
P = 0.46
PD-L1(CD274) expression
S NS
PD-L1(CD274)
Low(14)
High(13)
Proability
log-rank P = 0.88
OS (months)
C
IMvigor210 metastatic urothelial carcinoma immunotherapy cohort
P = 0.10
PD-L1(CD274) expression
S NS
PD-L1(CD274)
Low(177)
High(121)
Proability
log-rank P = 0.071
OS (months)
D
MSK-IMPACT cohort
P < 0.0001
NS (29)
S (15)
Proability
OS (Months)
MSK-IMPACT cohort
P < 0.0001
NS (29)
S (15)
PFS (Months)
E
MSK-IMPACT cohort (TMB)
P = 0.494
High (22)
Low (22)
OS (Months)
MSK-IMPACT cohort (TMB)
P = 0.454
High (22)
Low (22)
PFS (Months)
F
TCGA-ESCC cohort TMB
P = 0.835
High (44)
Low (45)
OS (Months)
G
HEK293T control experiments
Average pearson's correlation: 0.956
ESCC sample pool control experiments
Average pearson's correlation: 0.993
H
Proteins
784 9,310 923
S NS
Phosphoproteins
657 4,577 821
S NS
Phosphosites
7,248 24,726 8,555
S NS
P = 0.324
Number of proteins
P = 0.704
Number of phosphoproteins
P = 0.621
Number of phosphosites
I
Proteins
log10 (FOT)
Phosphoproteins
log10 (FOT)
Samples
J
Proteins
PC2 (9.8%)
PC1 (14.2%)
Phosphoproteins
PC2 (6.8%)
PC1 (13.8%)
K
PCA for MS detection batch effect
Batch−related information of the proteomic data: 6.44%
Spearman correlation
Explain ratio
Principal component
PCA for MS detection batch effect
Batch−related information of the phosphoproteomic data: 7.23%
Spearman correlation
Explain ratio
Principal component

 © The Author(s)

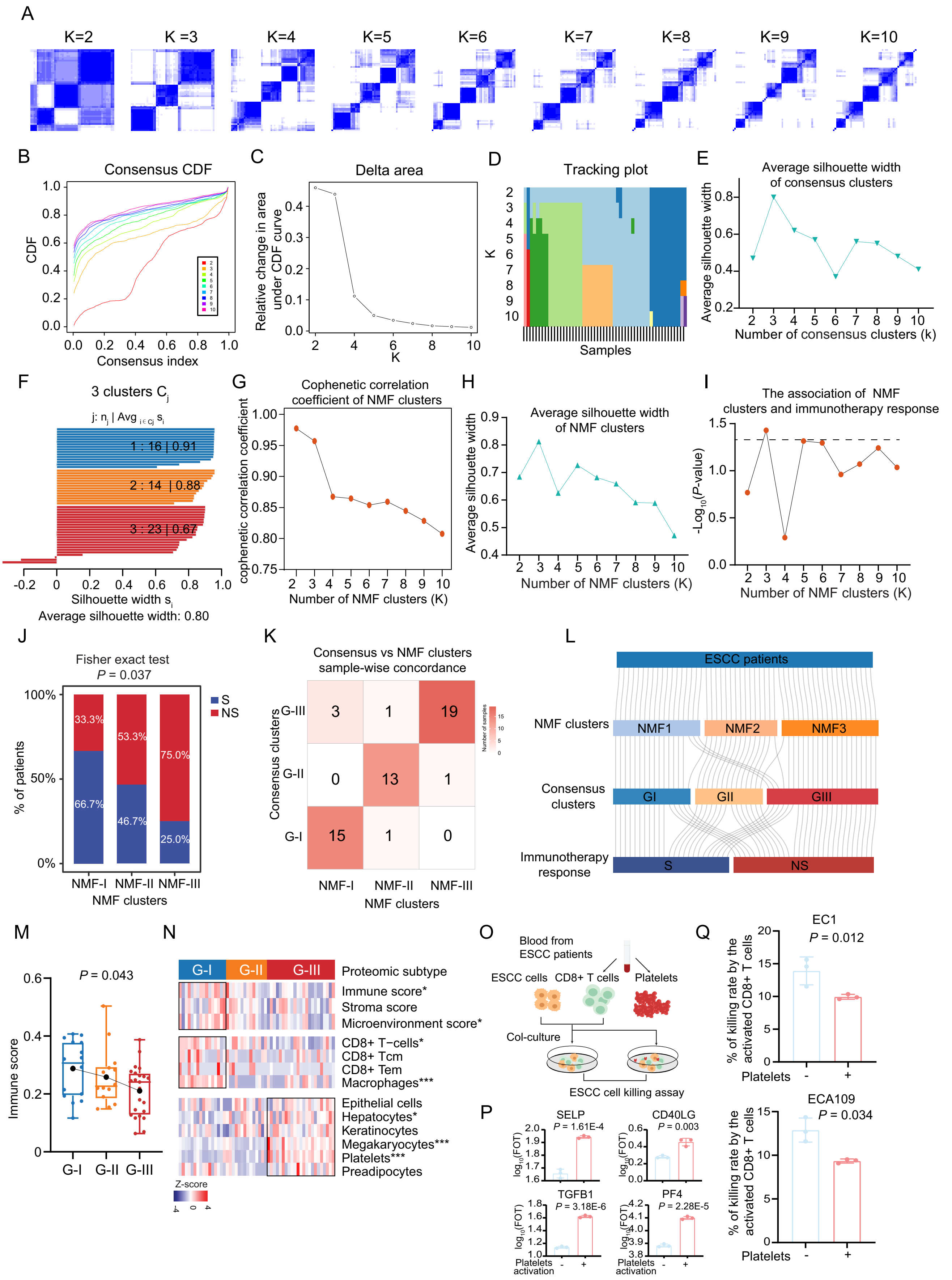
A
K=2
K =3
K=4
K=5
K=6
K=7
K=8
K=9
K=10
B
Consensus CDF
CDF
Consensus index
C
Delta area
Relative change in area under CDF curve
K
D
Tracking plot
Samples
E
Average silhouette width of consensus clusters
Average silhouette width
Number of consensus clusters (k)
F
3 clusters C_j
1 : 16 | 0.91
2 : 14 | 0.88
3 : 23 | 0.67
Silhouette width s_i
Average silhouette width: 0.80
G
Cophenetic correlation coefficient of NMF clusters
Number of NMF clusters (K)
H
Average silhouette width of NMF clusters
I
The association of NMF clusters and immunotherapy response
-Log10(P-value)
J
Fisher exact test
P = 0.037
% of patients
S
NS
NMF-I NMF-II NMF-III
NMF clusters
K
Consensus vs NMF clusters sample-wise concordance
Consensus clusters
G-III
G-II
G-I
L
ESCC patients
NMF clusters
NMF1
NMF2
NMF3
Consensus clusters
GI
GII
GIII
Immunotherapy response
S
NS
M
P = 0.043
Immune score
N
Proteomic subtype
Immune score*
Stroma score
Microenvironment score*
CD8+ T−cells*
CD8+ Tcm
CD8+ Tem
Macrophages***
Epithelial cells
Hepatocytes*
Keratinocytes
Megakaryocytes***
Platelets***
Preadipocytes
Z-score
O
Blood from ESCC patients
ESCC cells
CD8+ T cells
Platelets
Col-culture
ESCC cell killing assay
P
SELP P = 1.61E-4
CD40LG P = 0.003
TGFB1 P = 3.18E-6
PF4 P = 2.28E-5
Platelets activation
Q
EC1 P = 0.012
ECA109 P = 0.034
% of killing rate by the activated CD8+ T cells
Platelets

Figure EV2. Consensus clustering analysis of the ESCC immunotherapy cohort identified three proteomic subtypes.

(**A–D**) The consensus clustering analysis of 53 ESCC samples and three subtypes was generated. *K* was tested from 2 to 10 (**A**). Consensus matrices, as well as the consensus cumulative distribution function (CDF) plot (**B**), delta area (change in CDF area) plot (**C**), and tracking plot (**D**) are shown. (**E**) Average silhouette-width of the consensus subtypes ($K = 2$–10). The average silhouette-width takes the maximum value when the number of subtypes is 3 ($K = 3$). (**F**) The silhouette-width plot of three clusters. (**G**) Cophenetic correlation coefficient for the different choices of clusters in the non-negative matrix factorization (NMF) clustering. (**H**) The average silhouette-width score in different choices of clusters in NMF clustering. (**I**) The association of NMF clusters and ESCC immunotherapy response (*P* value from two-sided Fisher's exact test). (**J**) Barplot shows the distribution of immunotherapy response (S/NS) at the optimal choice of NMF clusters ($K = 3$). *P* value from two-sided Fisher's exact test. (**K**) Comparison of sample overlap in subtype assignment between consensus clusters and NMF clusters. (**L**) Sankey diagram indicates the comparison of NMF clusters and consensus clusters. (**M**) Boxplot for immune score among three proteomic subtypes (ANOVA test). Boxplots show median (central line), upper and lower quartiles (box limits), and minimum and maximum (whiskers). $n = 16$ (G-I), 14 (G-II), and 23 (G-IV). (**N**) Heatmap illustrating the dominant cell type compositions of G-I and G-III subtypes (two-sided Wilcoxon rank-sum test). *$P < 0.05$, **$P < 0.01$, ***$P < 0.001$, ****$P < 0.0001$. (**O**) Schematic illustrating the experimental design. (**P**) Barplots showing the molecular perturbation related to activated platelets ($n = 3$ independent experiments, two-sided Student's *t* test, mean with standard deviation). (**Q**) The influence of activated platelets on CD8 + T cell-mediated killing against ESCC cells ($n = 3$ independent experiments, two-sided Student's *t* test, mean with standard deviation).

 © The Author(s)

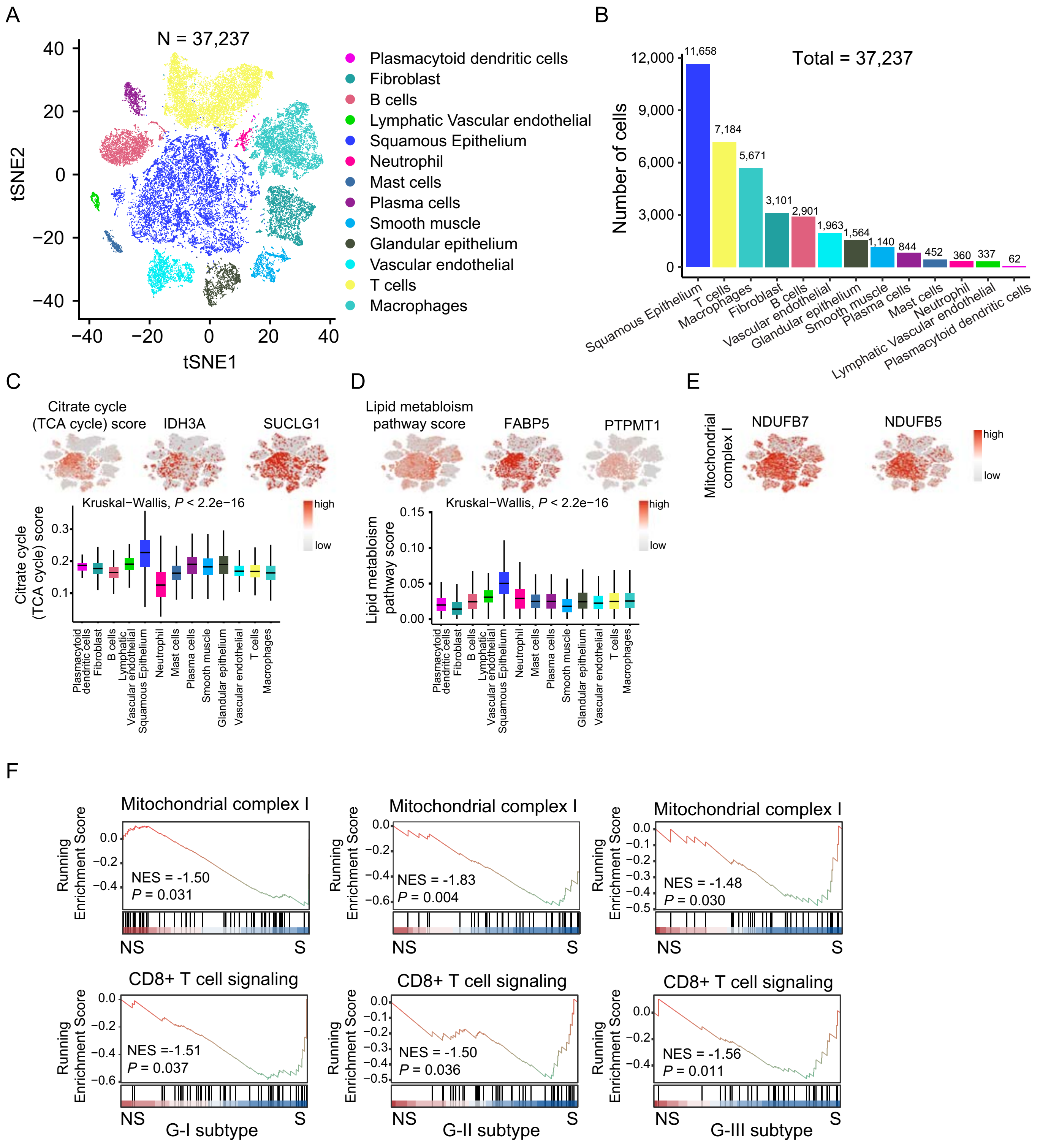
A
N = 37,237
tSNE2
tSNE1
Plasmacytoid dendritic cells
Fibroblast
B cells
Lymphatic Vascular endothelial
Squamous Epithelium
Neutrophil
Mast cells
Plasma cells
Smooth muscle
Glandular epithelium
Vascular endothelial
T cells
Macrophages
B
Total = 37,237
Number of cells
11,658
7,184
5,671
3,101
2,901
1,963
1,564
1,140
844
452
360
337
62
C
Citrate cycle (TCA cycle) score
IDH3A
SUCLG1
Kruskal–Wallis, P < 2.2e−16
Citrate cycle (TCA cycle) score
high
low
D
Lipid metabloism pathway score
FABP5
PTPMT1
Kruskal–Wallis, P < 2.2e−16
Lipid metabloism pathway score
E
Mitochondrial complex I
NDUFB7
NDUFB5
F
Mitochondrial complex I
Running Enrichment Score
NES = -1.50
P = 0.031
NES = -1.83
P = 0.004
NES = -1.48
P = 0.030
NS
S
CD8+ T cell signaling
NES =-1.51
P = 0.037
NES = -1.50
P = 0.036
NES = -1.56
P = 0.011
G-I subtype
G-II subtype
G-III subtype

Figure EV3. The scRNA-seq analysis of ESCC from Pan et al.

(**A**) The t-SNE map of the single-cell ESCC landscape was colored by major cell subtypes. (**B**) The number of each cell subtype in the ESCC tumor microenvironment. (**C–E**) The t-SNE maps of individual cell AUC score overlay for selected pathway activity and the protein involved in the pathways (upper), and the boxplots show the AUC score of selected pathways in each cell subtype (lower) (two-sided Kruskal–Wallis test). Boxplots represent the interquartile range (IQR), with the box spanning the 25th to 75th percentiles and the median indicated by a horizontal line. Whiskers extend to the most extreme data points within 1.5×IQR. $n = 62$ (plasmacytoid dendritic cells), 3101 (fibroblast), 2901 (B cells), 337 (lymphatic vascular endothelial), 11,658 (squamous epithelium), 360 (neutrophil), 452 (mast cells), 844 (plasma cells), 1140 (smooth muscle), 1564 (glandular epithelium), 1963 (vascular endothelial), 7184 (T cells), and 5671 (macrophages). (**F**) Pathway enrichment of mitochondrial complex I and CD8 + T cell signaling pathways between S and NS groups in three proteomic subtypes (phenotype-based permutation test).

 © The Author(s)

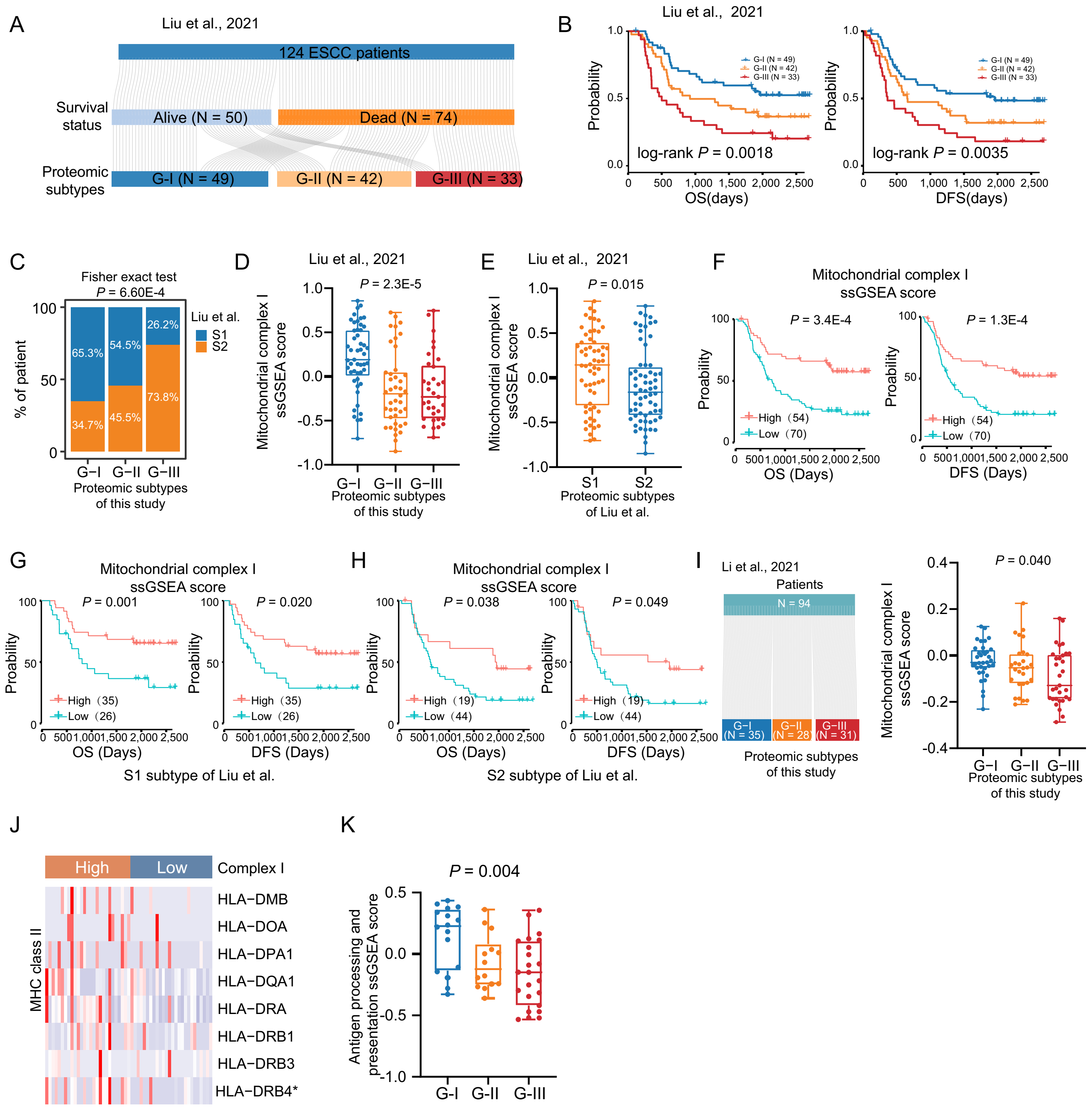
A
Liu et al., 2021
124 ESCC patients
Survival status
Alive (N = 50)
Dead (N = 74)
Proteomic subtypes
G-I (N = 49)
G-II (N = 42)
G-III (N = 33)
B
Liu et al., 2021
Probability
log-rank P = 0.0018
OS(days)
log-rank P = 0.0035
DFS(days)
C
Fisher exact test
P = 6.60E-4
% of patient
Liu et al.
S1
S2
Proteomic subtypes of this study
D
Liu et al., 2021
P = 2.3E-5
Mitochondrial complex I ssGSEA score
Proteomic subtypes of this study
E
Liu et al., 2021
P = 0.015
Proteomic subtypes of Liu et al.
F
Mitochondrial complex I ssGSEA score
P = 3.4E-4
P = 1.3E-4
Proabability
High (54)
Low (70)
OS (Days)
DFS (Days)
G
Mitochondrial complex I ssGSEA score
P = 0.001
P = 0.020
High (35)
Low (26)
S1 subtype of Liu et al.
H
Mitochondrial complex I ssGSEA score
P = 0.038
P = 0.049
High (19)
Low (44)
S2 subtype of Liu et al.
I
Li et al., 2021
Patients
N = 94
G-I (N = 35)
G-II (N = 28)
G-III (N = 31)
Proteomic subtypes of this study
P = 0.040
J
High
Low
Complex I
MHC class II
HLA-DMB
HLA-DOA
HLA-DPA1
HLA-DQA1
HLA-DRA
HLA-DRB1
HLA-DRB3
HLA-DRB4*
K
P = 0.004
Antigen processing and presentation ssGSEA score
G-I G-II G-III

Figure EV4. The validation of proteomics subtypes in other ESCC cohorts.

(**A**) Clustering of 124 ESCC patients using the proteomic data from Liu et al by the proteomic subtyping algorithm of this study. (**B**) Survival analysis (OS and DFS) of ESCC patients from Liu et al classified by the proteomic subtyping algorithm of this study (*P* value from two-sided log-rank test). (**C**) Barplot showing the S1 and S2 subtypes of Liu et al distribution among the three proteomic subtypes of this study (two-sided Fisher's exact test). (**D**) Boxplots showing mitochondrial complex I ssGSEA score across the three proteomic subtypes in the study of Liu et al (ANOVA test). Boxplots represent the interquartile range (IQR), with the box spanning the 25th to 75th percentiles and the median indicated by a horizontal line. Whiskers mark minimum or maximum values. $n = 49$ (G-I), 42 (G-II), and 33 (G-III). (**E**) Boxplots showing mitochondrial complex I ssGSEA score between S1 ($n = 61$) and S2 ($n = 63$) subtypes from Liu et al (two-sided Student's *t* test). Boxplots are defined as in (**D**). (**F**) Survival analysis (OS and DFS) of mitochondrial complex I ssGSEA score in the study of Liu et al (two-sided log-rank test). (**G**, **H**) Survival analysis (OS and DFS) of mitochondrial complex I ssGSEA score in the S1 and S2 subtypes identified by Liu et al, respectively (two-sided log-rank test). (**I**) The application of our proteomic subtypes in the study of Li et al. Boxplots displaying mitochondrial complex I ssGSEA score across the three proteomic subtypes in the study of Li et al (ANOVA test). Boxplots are defined as in (**D**). $n = 35$ (G-I), 28 (G-II), and 31 (G-III). (**J**) Differential expression of MHC class II proteins between mitochondrial complex I high and low groups (two-sided Wilcoxon rank-sum test). $^{*}P < 0.05$. (**K**) Boxplot shows the ssGSEA score of the antigen processing and presentation pathway among three proteomic subtypes (ANOVA test). Boxplots are defined as in (**D**). $n = 16$ (G-I), 14 (G-II), and 23 (G-III).

© The Author(s)

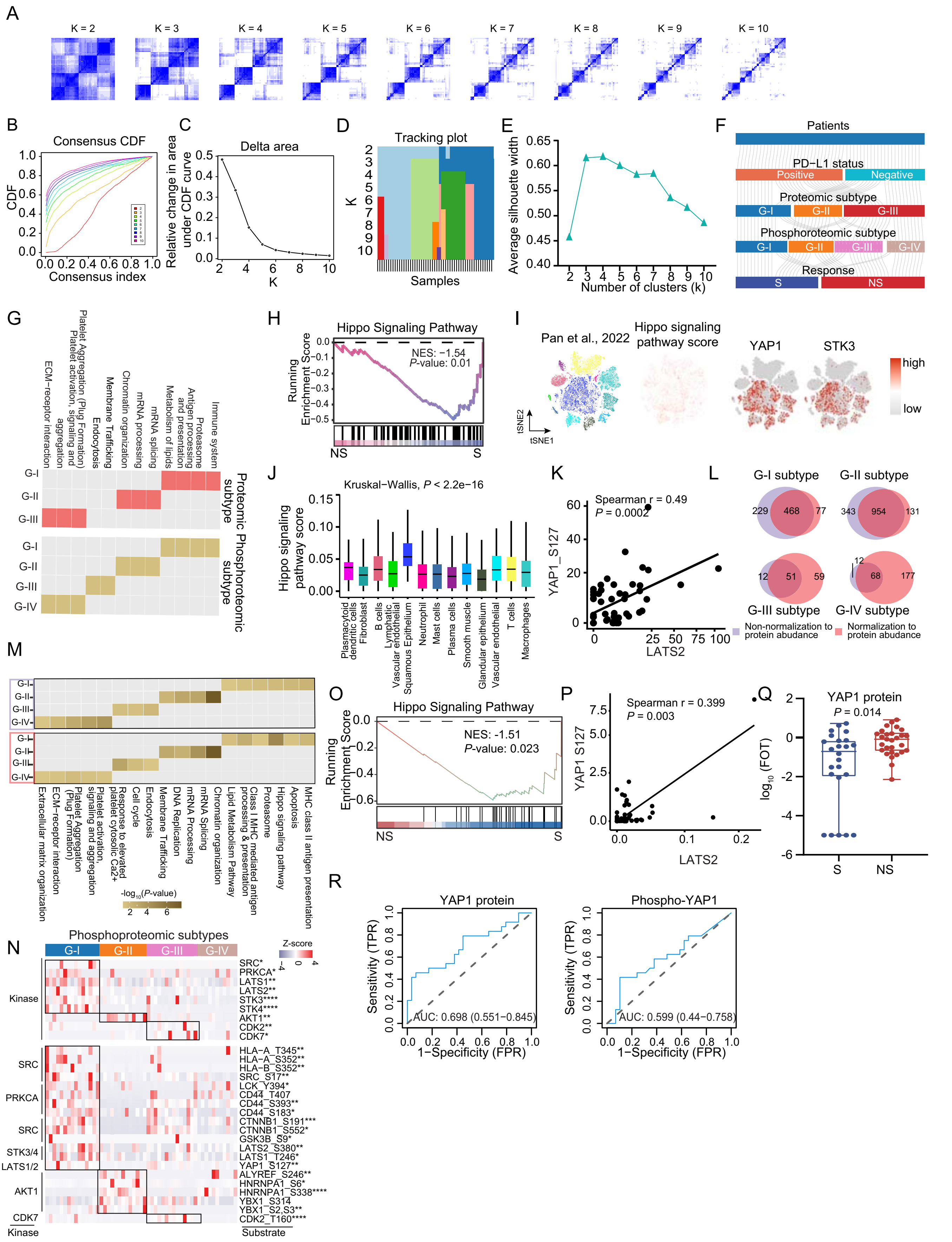

A
K = 2
K = 3
K = 4
K = 5
K = 6
K = 7
K = 8
K = 9
K = 10
B
Consensus CDF
CDF
Consensus index
C
Delta area
Relative change in area under CDF curve
K
D
Tracking plot
Samples
E
Average silhouette width
Number of clusters (k)
F
Patients
PD−L1 status
Positive
Negative
Proteomic subtype
Phosphoroteomic subtype
Response
G
Proteomic subtype
Phosphoroteomic subtype
H
Hippo Signaling Pathway
Running Enrichment Score
NES: −1.54
P-value: 0.01
I
Pan et al., 2022
Hippo signaling pathway score
YAP1
STK3
high
low
J
Kruskal−Wallis, P < 2.2e−16
Hippo signaling pathway score
K
Spearman r = 0.49
P = 0.0002
YAP1_S127
LATS2
L
G-I subtype
G-II subtype
G-III subtype
G-IV subtype
Non-normalization to protein abudance
Normalization to protein abudance
M
-log10(P-value)
O
Hippo Signaling Pathway
NES: -1.51
P-value: 0.023
P
Spearman r = 0.399
P = 0.003
YAP1 S127
LATS2
Q
YAP1 protein
P = 0.014
log10 (FOT)
R
YAP1 protein
Phospho-YAP1
Sensitivity (TPR)
1−Specificity (FPR)
AUC: 0.698 (0.551−0.845)
AUC: 0.599 (0.44−0.758)
N
Phosphoproteomic subtypes
Z-score
Kinase
Substrate

Figure EV5. eusoft-did-translate-comment-enPhosphoproteomic subtyping of ESCC immunotherapy cohort and correlation with immunotherapy response.

(**A–D**) The consensus clustering analysis of 53 ESCC samples and four subtypes was generated. *K* was tested from 2 to 10 (**A**). Consensus matrices, as well as the consensus cumulative distribution function (CDF) plot (**B**), delta area (change in CDF area) plot (**C**), and tracking plot (**D**) are shown. (**E**) Average silhouette-width of identified subtypes ($K = 2$–10). The average silhouette width takes the maximum value at the 4 subtypes ($K = 4$). (**F**) Sankey plot showing the flow of the ESCC patients featured with different PD-L1 expression status to proteomic subtypes, phosphoproteomic subtypes, and immunotherapy response groups. (**G**) The comparison of pathway enrichment between proteomic subtypes and phosphoproteomic subtypes. (**H**) GSEA enrichment plot of the hippo signaling pathway in S and NS groups at the phosphoproteome level. *P* value from phenotype-based permutation test. (**I**) The t-SNE maps of individual cell AUC score overlay for hippo signaling pathway score using the scRNA-seq data from Pan et al, and the protein involved in the hippo signaling pathway. (**J**) The boxplots show the AUC score of the hippo signaling pathway in each cell cluster (two-sided Kruskal–Wallis test). Boxplots represent the interquartile range (IQR), with the box spanning the 25th to 75th percentiles and the median indicated by a horizontal line. Whiskers extend to the most extreme data points within 1.5×IQR. $n = 62$ (plasmacytoid dendritic cells), 3101 (fibroblast), 2901 (B cells), 337 (lymphatic vascular endothelial), 11,658 (squamous epithelium), 360 (neutrophil), 452 (mast cells), 844 (plasma cells), 1140 (smooth muscle), 1564 (glandular epithelium), 1963 (vascular endothelial), 7184 (T cells), and 5671 (macrophages). (**K**) Spearman correlation of LATS2 and its phosphorylated substrate YAP1 S127. *P* value was from a two-sided Spearman's correlation test. (**L**) The overlap of significant expression phosphoproteins with or without normalization to the corresponding protein abundance in each phosphoproteomic subtype. (**M**) Comparison of pathway enrichment with or without normalization to the corresponding protein abundance.
(**N**) Heatmap showing the kinases and their substrates in each phosphoproteomic subtype after normalization (two-sided Wilcoxon rank-sum test). $^{*}P < 0.05$, $^{**}P < 0.01$, $^{***}P < 0.001$, $^{****}P < 0.0001$. (**O**) GSEA enrichment plot of the hippo signaling pathway in S and NS groups at the phosphoproteome level after normalization. *P* value from phenotype-based permutation test. (**P**) Spearman correlation of LATS2 and its phosphorylated substrate YAP1 S127 after normalization to its protein abundance. *P* value was from two-sided Spearman's correlation test. (**Q**) Boxplot showing the YAP1 protein expression in S ($n = 24$) and NS ($n = 29$) patients (two-sided Wilcoxon rank-sum test). Boxplots represent the interquartile range (IQR), with the box spanning the 25th to 75th percentiles and the median indicated by a horizontal line. Whiskers mark minimum or maximum values. (**R**) Area under receiver operating characteristic (ROC) curves of YAP1 protein and phosphorylation in the prediction of ESCC immunotherapy response.

 © The Author(s)

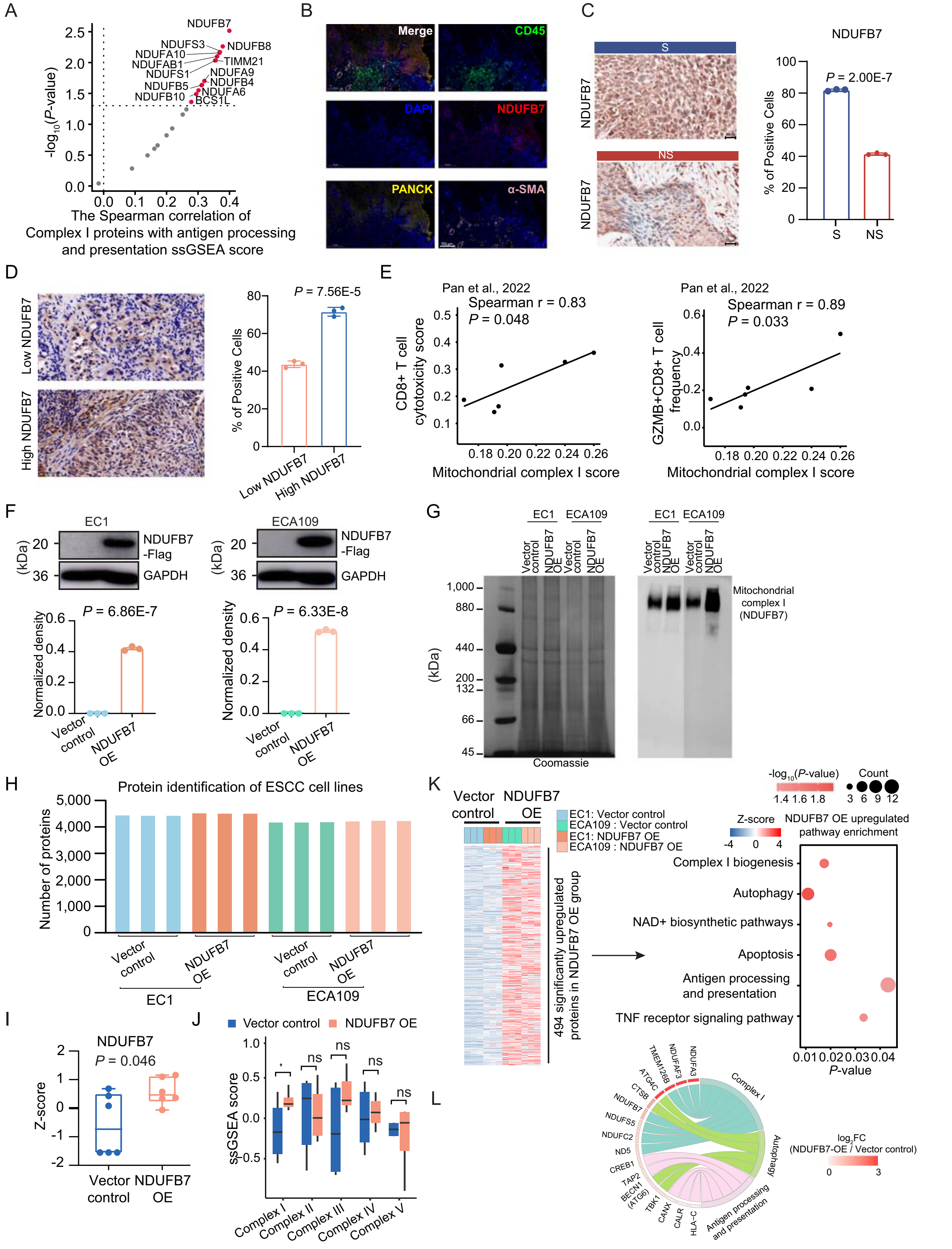
A
NDUFB7
NDUFS3
NDUFB8
NDUFA10
NDUFAB1
TIMM21
NDUFS1
NDUFA9
NDUFB5
NDUFB4
NDUFA6
NDUFB10
BCS1L
-log10(P-value)
The Spearman correlation of Complex I proteins with antigen processing and presentation ssGSEA score
B
Merge
CD45
DAPI
NDUFB7
PANCK
α-SMA
C
S
NS
NDUFB7
P = 2.00E-7
% of Positive Cells
D
Low NDUFB7
High NDUFB7
P = 7.56E-5
% of Positive Cells
E
Pan et al., 2022
Spearman r = 0.83
P = 0.048
CD8+ T cell cytotoxicity score
Mitochondrial complex I score
Pan et al., 2022
Spearman r = 0.89
P = 0.033
GZMB+CD8+ T cell frequency
Mitochondrial complex I score
F
EC1
ECA109
NDUFB7 -Flag
GAPDH
(kDa)
P = 6.86E-7
P = 6.33E-8
Normalized density
Vector control
NDUFB7 OE
G
EC1
ECA109
Vector control
NDUFB7 OE
(kDa)
Coomassie
Mitochondrial complex I (NDUFB7)
H
Protein identification of ESCC cell lines
Number of proteins
Vector control
NDUFB7 OE
EC1
ECA109
I
NDUFB7
P = 0.046
Z-score
Vector control
NDUFB7 OE
J
Vector control
NDUFB7 OE
ssGSEA score
ns
Complex I
Complex II
Complex III
Complex IV
Complex V
K
Vector control
NDUFB7 OE
EC1: Vector control
ECA109 : Vector control
EC1: NDUFB7 OE
ECA109 : NDUFB7 OE
494 significantly upregulated proteins in NDUFB7 OE group
-log10(P-value)
Count
Z-score
NDUFB7 OE upregulated pathway enrichment
Complex I biogenesis
Autophagy
NAD+ biosynthetic pathways
Apoptosis
Antigen processing and presentation
TNF receptor signaling pathway
P-value
L
Complex I
Autophagy
Antigen processing and presentation
log2FC (NDUFB7-OE / Vector control)
NDUFA3
NDUFAF3
TMEM126B
ATG4C
CTSB
NDUFB7
NDUFS5
NDUFC2
ND5
CREB1
TAP2
BECN1 (ATG6)
TBK1
CANX
CALR
HLA-C

Figure EV6. A possible mechanism by which mitochondrial complex I modulates immunotherapy sensitivity via CD8 + T cell-mediated killing in vitro.

(**A**) Volcano showing the Spearman correlation of mitochondrial complex I proteins with antigen processing and presentation ssGSEA score (two-sided Spearman's correlation test). (**B**) Representative images of multiplex immunofluorescence staining for NDUFB7, PANCK, CD45, and α-SMA in ESCC biopsy tissues. (**C**) The representative images of immunohistochemistry (IHC) staining of NDUFB7 expression in sensitive (S) and non-sensitive (NS) groups. Boxplot showing the qualification of NDUFB7 stained by immunohistochemistry (IHC) in the representative samples in the S and NS groups ($n = 3$ independent experiments, two-sided Student's t test, mean with standard deviation). Scale bar: 20 μm. (**D**) ESCC tumor with different expression of NDUFB7 determined by IHC ($n = 3$ independent experiments, two-sided Student's t test, mean with standard deviation). (**E**) Correlation between mitochondrial complex I score and CD8 + T cells cytotoxicity score or GZMB + CD8 + T cell frequency in ESCC patients in scRNA-seq data of Pan et al (two-sided Spearman correlation test). (**F**) Immunoblot of NDUFB7 (tagged with a Flag) and GAPDH in EC1 and ECA109 cells, and the normalization of qualified western blots ($n = 3$ independent experiments, two-sided Student's t test, mean with standard deviation). (**G**) Analysis of mitochondrial complex I by BN-PAGE and immunoblotting in NDUFB7 OE ESCC cells. Coomassie staining was used as a loading control. (**H**) Barplots showing the numbers of identified proteins in EC1 and ECA109 cells with overexpression of NDUFB7 or vector control. (**I**) Boxplots showing the differential expression of NDUFB7 between NDUFB7 OE ($n = 6$) and vector control ($n = 6$) groups (two-sided Student's t test). Boxplots represent the interquartile range (IQR), with the box spanning the 25th to 75th percentiles and the median indicated by a horizontal line. Whiskers mark minimum or maximum values. (**J**) Boxplot of the multiple mitochondrial complexes ssGSEA score between NDUFB7 OE ($n = 6$) and vector control ($n = 6$) groups (two-sided Wilcoxon rank-sum test). Boxplots are defined as in (**I**). $P = 0.047$ (Complex I), 0.0944 (Complex II), 0.098 (Complex III), 0.333 (Complex IV), and 0.918 (Complex V). *$P < 0.05$, ns not significant. (**K**) Heatmap showing the differential expression of the overrepresented proteins in the NDUFB7 OE ESCC cells (left). The bubble plot shows the significantly enriched pathways in NDUFB7 OE ESCC cells (right) (hypergeometric test). (**L**) Circular plot showing differentially expressed proteins involved in mitochondrial complex I, autophagy, and antigen processing and presentation pathways between NDUFB7 OE and vector control groups.

 © The Author(s)

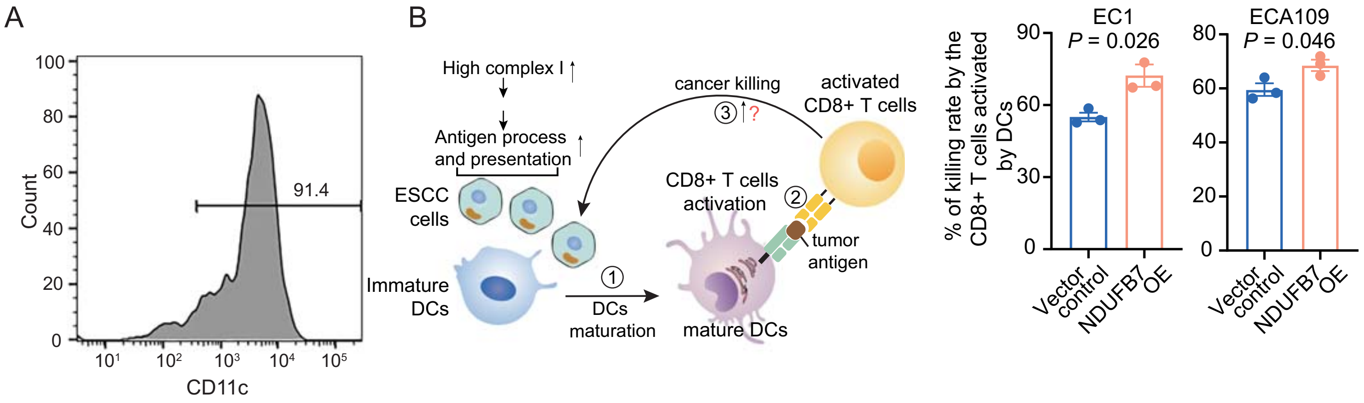

Figure EV7. Mitochondrial complex I increases the anti-tumor ability of CD8 + T cells activated by dendritic cells.

(**A**) Flow cytometry histogram representing the percentage of DCs isolated from PBMC measured by their surface markers CD11c. (**B**) Diagram showing the co-cultured system composed of ESCC cells, DCs, and activated CD8 + T cells. Boxplots showing the killing effect of CD8 + T cells activated by DCs co-cultured with NDUFB7 OE ESCC cells and vector controls ($n = 3$ independent experiments, two-sided Student's t test, mean with standard deviation).

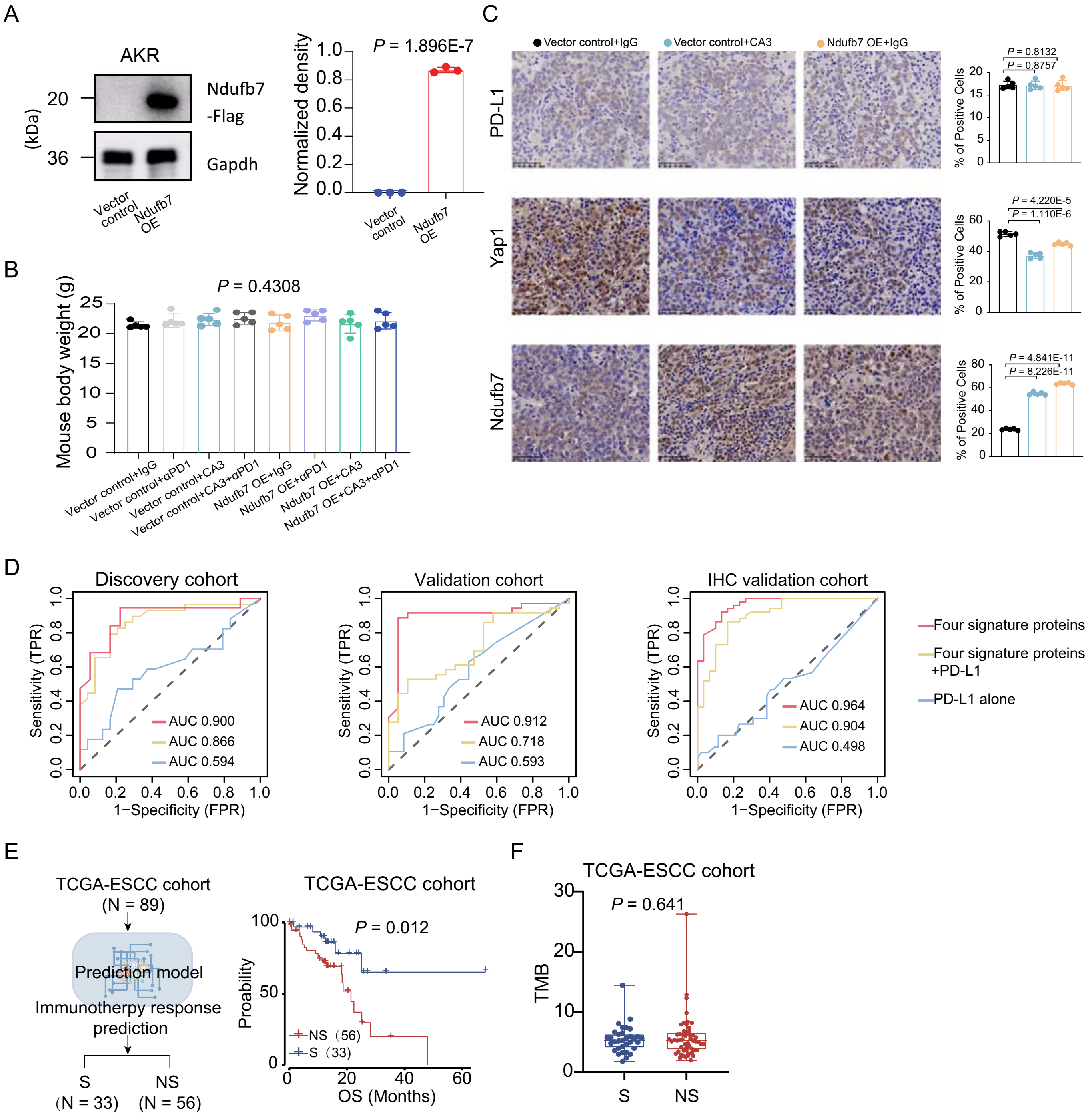

Figure EV8. The mitochondrial complex I of ESCC increases the therapeutic efficacy of anti-PD1 treatment in vivo.

(**A**) Immunoblot analysis of Ndufb7 (tagged with a Flag) and Gapdh in mouse ESCC cell AKR, and the normalization of qualified western blots ($n = 3$ independent experiments, two-sided Student's t test, mean with standard deviation). (**B**) The mouse body weights in the AKR allografts model ($n = 5$/group, ANOVA test, mean with standard deviation). (**C**) Representative IHC staining images and the quantification of the positive cells of PD-L1, Ndufb7, and Yap1 in the vector control tumor, vector control tumor with CA3 treatment, and Ndufb7 OE tumor ($n = 5$/group, ANOVA test, mean with standard deviation). (**D**) The ROC curves of the four signature proteins with or without the combination of PD-L1 in the discovery cohort, validation cohort, and IHC validation cohort, respectively. (**E**) The predicted result of immunotherapy response for ESCC patients in the TCGA-ESCC cohort using our predictive model. (**F**) Boxplots showing the differences of TMB level between S ($n = 33$) and NS ($n = 56$) patients in the TCGA-ESCC cohort (two-sided Student's t test). Boxplots represent the interquartile range (IQR), with the box spanning the 25th to 75th percentiles and the median indicated by a horizontal line. Whiskers mark minimum or maximum values.

 © The Author(s)

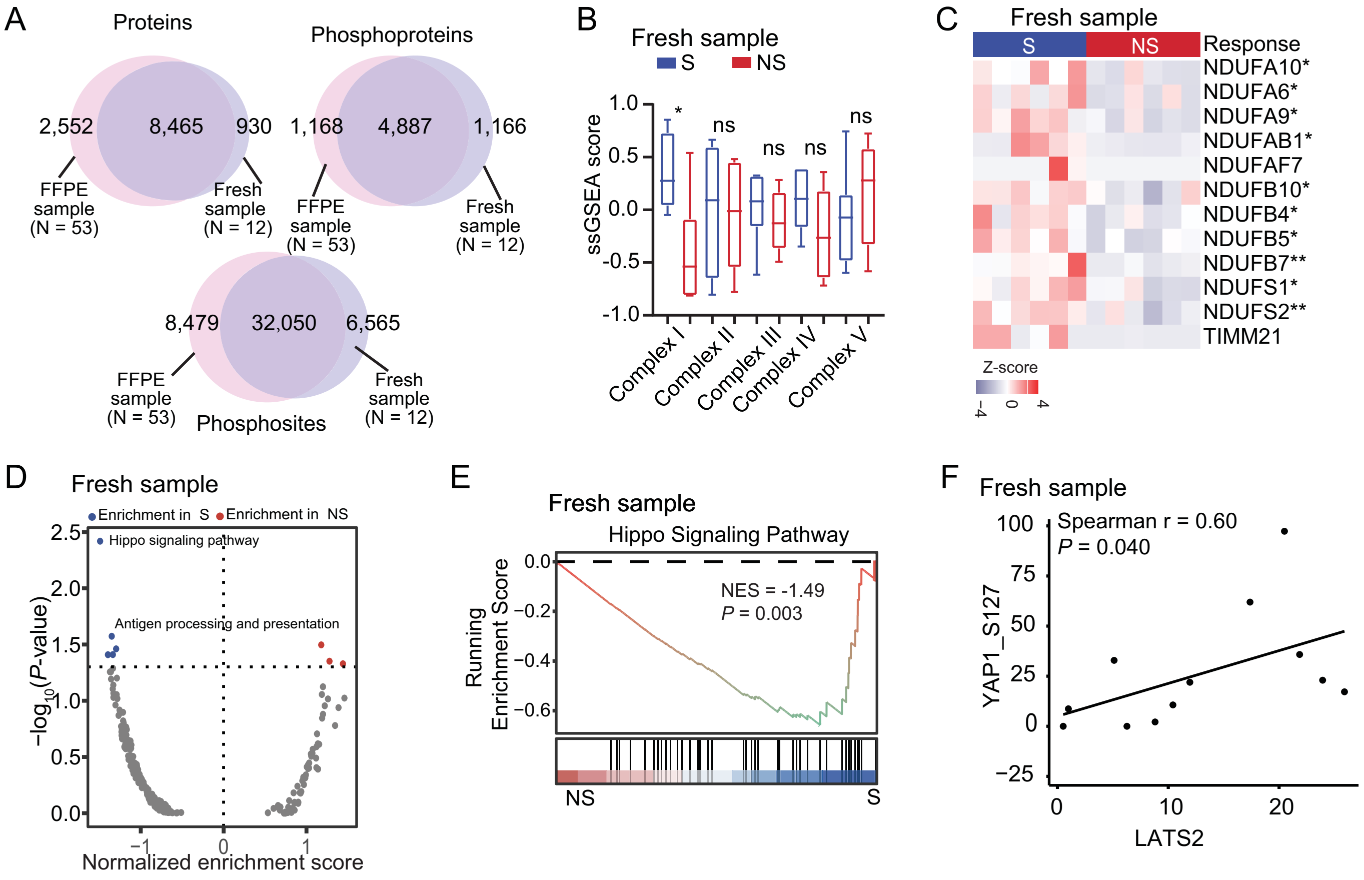

Figure EV9. The comparative results of proteomic and phosphoproteomic analysis from fresh tissues.

(**A**) The comparison of protein, phosphoprotein, and phosphosite identification between FFPE and fresh tissues. (**B**) The ssGSEA score of mitochondrial complexes I–V between S ($n = 6$) and NS ($n = 6$) patients with fresh tissues. *P* value from two-sided Wilcoxon rank-sum test. Boxplots represent the interquartile range (IQR), with the box spanning the 25th to 75th percentiles and the median indicated by a horizontal line. Whiskers mark minimum or maximum values. $P = 0.026$ (Complex I), 0.937 (Complex II), 0.485 (Complex III), 0.240 (Complex IV), and 0.481 (Complex V). *$P < 0.05$, ns not significant. (**C**) Heatmap showing mitochondrial complex I proteins in S and NS patients with fresh tissues (two-sided Wilcoxon rank-sum test). *$P < 0.05$, **$P < 0.01$, ***$P < 0.001$. (**D**) Volcano plot showing the pathway enrichment between S and NS groups based on the phosphoproteome by GSEA analysis in fresh tissues. *P* value from Phenotype-based permutation test. (**E**) GSEA enrichment plot of the hippo signaling pathway in S patients in fresh tissues. *P* value from Phenotype-based permutation test. (**F**) Spearman correlation of LATS2 and its phosphorylated substrate YAP1 S127 in fresh tissues. *P* value was from two-sided Spearman's correlation test.

© The Author(s)
